# Gut Microbiome Differences Across Mixed-Sex and Female-Only Social Rearing Regimes in Female Field Crickets *Teleogryllus occipitalis* (Orthoptera: Gryllidae)

**DOI:** 10.3390/insects17010091

**Published:** 2026-01-13

**Authors:** Kazuya Hirata, Takeshi Suzuki, Kei Yura, Toru Asahi, Kosuke Kataoka

**Affiliations:** 1 Graduate School of Advanced Science and Engineering, Waseda University, Tokyo 169-8555, Japan; 2 Graduate School of Bio-Applications and Systems Engineering, Tokyo University of Agriculture and Technology, Tokyo 184-8588, Japan; 3 Graduate School of Humanities and Sciences, Ochanomizu University, Tokyo 112-8610, Japan; 4 Department of Life Science and Medical Bioscience, Waseda University, Tokyo 162-8480, Japan; 5 Comprehensive Research Organization, Waseda University, Tokyo 162-0041, Japan; 6 Graduate School of Engineering, Tokyo University of Agriculture and Technology, Tokyo 184-8588, Japan

**Keywords:** gut microbiome, whole-genome shotgun sequencing, field cricket, social rearing regime, *Teleogryllus occipitalis*

## Abstract

The insect gut microbiome contributes to various host physiological processes and behaviors, such as digestion, nutrient absorption, immunity, mate choice, and fecundity. The social environment can shape gut microbial communities. Mixed-sex vs. female-only rearing is an important social context because it differs in exposure to the opposite sex and mating opportunities, which may in turn affect female physiology that may influence their gut microbiome. Despite the growing recognition of these social-microbial interactions, most studies have relied on 16S rRNA amplicon sequencing or qPCR, which provide only coarse taxonomic resolution and limited functional insight. In this study, we used whole-genome shotgun metagenomics to examine changes in microbial diversity and functional gene composition in the female field cricket *Teleogryllus occipitalis* (Serville) (Orthoptera: Gryllidae) reared under two social conditions: mixed-sex rearing and female-only rearing. Species richness and diversity analyses revealed that community composition separated between females from mixed-sex and female-only rearing. Functional profiling indicated higher relative abundances of genes annotated to nutrient processing and inter-bacterial competition in females from mixed-sex rearing, whereas females from female-only rearing showed relative enrichment of genes annotated to stress resistance and nitrogen fixation. These findings provide a genome-resolved foundation for testing how social rearing conditions covary with gut microbiome composition and functional potential in female crickets.

## Simple Summary

Crickets are useful model organisms with incomplete metamorphosis for understanding insect physiology and behavior. Like all animals, they host a gut microbiome, a community of tiny organisms that helps digest food, fight disease, and support reproduction. Social environment, such as being reared under mixed-sex versus female-only group-rearing regimes, may change the female gut microbiome, but this has rarely been tested in insects using genetic approaches. We studied the field cricket *Teleogryllus occipitalis* and compared the gut microbiome of females in mixed-sex rearing with that of females in female-only rearing. Using whole-genome shotgun metagenomics, we found consistent differences in both microbial composition and gene content between the two rearing regimes. Females from mixed-sex rearing showed higher relative abundances of microbial genes annotated to breaking down complex nutrients and competing with other microbes, whereas females from female-only rearing showed more genes annotated to stress tolerance and coping with limited nutrition. These findings suggest that the social rearing regime (mixed-sex vs. female-only) is associated with the gut microbiome of female crickets.

## 1. Introduction

Insects harbor symbiotic gut microbiomes that influence a wide range of behavioral and physiological processes in their hosts, such as food digestion that supports growth and development [1], supplementation of nutritionally deficient diets [2], immune regulation [3], mate selection [4], and reproductive performance [5].

The social environment that an animal experiences can shape its gut microbiome, potentially influencing host behavior and physiology. In particular, mixed-sex versus same-sex social contexts, which differ in exposure to the opposite sex and mating opportunities, have emerged as important factors, and in many species, females show pronounced physiological responses to these environments. For instance, in the dengue mosquito *Aedes aegypti* (Linnaeus), mating suppresses female immunity, promotes bacterial proliferation, and ultimately enhances egg production [6]. In the fall armyworm *Spodoptera frugiperda* (Smith), mating reduces the alpha-diversity of gut microbial communities [7]. Virgin females of the Mormon cricket *Anabrus simplex* (Haldeman) that are deprived of male contact exhibit a decline in beneficial lactic acid bacteria [8], whereas females of the fruit fly *Drosophila suzukii* (Matsumura) can horizontally acquire male-derived microbes during mating [9]. However, most of these findings are based on 16S rRNA amplicon sequencing or qPCR, which provide limited taxonomic resolution and functional insight. In contrast, shotgun metagenomics enables comprehensive profiling of microbial gene content, metabolic pathways, and species-level taxonomic profiling [10], filling gaps in our understanding of how differences in the social environment are reflected in gut microbiome function. Therefore, comprehensive metagenomic approaches are needed to characterize microbiome gene content at higher resolution and generate hypotheses about the functional significance of microbiome variation associated with social context.

Crickets are valuable model organisms for studying social environments because of their pronounced sex-specific social roles [11]. Males produce acoustic signals for communication, whereas females exhibit distinct reproductive behaviors. Co-housing females with males markedly alters their physiology and behavior, including immune suppression, increased sexual responsiveness to males, increased body size, and changes in feeding behavior [12,13,14,15]. Our previous study based on 16S rRNA amplicon sequencing of the field cricket *Teleogryllus occipitalis* (Serville) (Orthoptera: Gryllidae) revealed sex-specific differences in gut microbiome, with females showing enrichment of predicted metabolic functions related to carbohydrate metabolism and energy production, potentially supporting egg development [16]. However, this characterization focused on static sex differences and did not address variation across social rearing regimes. Consequently, it remains unclear how the gut microbiome of female crickets differs between mixed-sex and female-only rearing environments.

In this study, we used whole-genome shotgun metagenomic sequencing to characterize the hindgut microbiome of female *T. occipitalis* in relation to the social rearing regimes. We primarily compared gut microbial communities between females from mixed-sex rearing and females from female-only rearing. Given evidence from our prior work suggesting that gut microbiomes may differ by sex [16], we also assessed female–male differences within the mixed-sex rearing condition for further investigation. To enable these female–male comparisons under the same rearing environment, male crickets were sampled from the mixed-sex cages. We did not include a male-only rearing treatment because it would address a separate question about male gut microbiome variation under single-sex rearing, which was beyond the scope of this study. Our genome-resolved metagenomic data document gut microbiome differences in female field crickets that are associated with social rearing regime (mixed-sex vs. female-only rearing).

## 2. Materials and Methods

### 2.1. Rearing Conditions, Gut Dissection, and DNA Extraction of T. occipitalis

A population of *T. occipitalis* was collected from Amami-Ōshima Island, Kagoshima Prefecture, Japan. The population was reared in plastic cages under a photoperiod of L:D = 16:8 h at 30 °C. In the cage, crickets had ad libitum access to commercial chicken feed (Chougenki Edzukeyousuuyou; Nosan Corp., Kanagawa, Japan) and water, and both were replaced with fresh supplies every 2–3 d. After the crickets reached the late-instar stage, when their sex could be distinguished, they were assigned to either mixed-sex cages (500 × 390 × 325 mm), where males and females were reared together until adulthood, or female-only cages, where females were reared in female-only groups until adulthood. In the mixed-sex cages, males and females interacted freely, and mating could occur. However, individual mating events, including mating status and mating frequency, were not monitored or experimentally controlled. Therefore, the mixed-sex treatment should be interpreted as a composite social rearing regime that includes male exposure and the possibility of mating. Throughout the rearing period, per-cage density was maintained at 30–50 individuals (0.015–0.026 individuals/cm^2^) for both treatments. Although density varied within this range, it remained below levels reported to cause adverse physiological effects in related species [17], making density-related stress less likely to act as a major confounder. To eliminate residual food and transient microbes, all adults were starved for 15–17 h before dissection, as previously described [18]. For the female samples, only individuals whose abdomens were filled with mature eggs (i.e., eggs comparable in size to those laid on the substrate) were selected, ensuring that all sampled females were ready to oviposit.

Females from mixed-sex rearing (*n* = 15), females from female-only rearing (*n* = 15), and male crickets from mixed-sex rearing (*n* = 15) were surface-sterilized in 70% ethanol. Males were included only as a reference for sex-related differences within the shared mixed-sex rearing environment, and a male-only rearing group was not part of the study design. To reduce potential confounding effects of group housing, such as injuries and contamination from cuticle-associated bacteria, we sampled only individuals without overt wounds. We then performed aseptic hindgut dissections after surface sterilization. The hindgut, which includes the ileum, colon, and rectum, was excised with flame-sterilized instruments, as described in our previous study [16]. To obtain a stable estimate of group-level microbiome composition while mitigating inter-individual variability, we randomly pooled five hindguts per sample with equal tissue input from each individual. Each pool underwent independent extraction and library preparation and was treated as an experimental unit.

DNA extraction was performed according to a previously described protocol [19] with slight modifications. Before DNA extraction, the homogenate was resuspended in 400 µL of 10 mM Tris-Cl (pH 8.0), thoroughly mixed with 100 µL of lysozyme solution, and combined with 500 µL of cell lysis buffer (10 mM Tris-HCl, 26 mM EDTA, 0.5% [*w*/*v*] SDS). After gentle agitation at 37 °C for approximately 45 min to allow sufficient dissolution, Proteinase K (50 µL) was added, and incubation proceeded at 56 °C for 30 min. To remove residual RNA, 15 µL of RNase A was added, followed by a 5 min incubation at 56 °C. Finally, genomic DNA was extracted using phenol/chloroform/isoamyl alcohol (25:24:1).

### 2.2. DNA Sequencing and Quality Control of Metagenomic Reads

The extracted DNA samples were shipped to Seibutsu Giken Inc. (Kanagawa, Japan), where next-generation sequencing libraries were prepared and subsequently sequenced. The concentration of extracted DNA was measured using a Synergy LX (Agilent Technologies, Santa Clara, CA, USA) and the QuantiFluor dsDNA System (Promega, Madison, WI, USA). Sequencing libraries were prepared using the MGIEasy FS DNA Library Prep Set (MGI Tech, Shenzhen, Guangdong, China) following the manufacturer’s instructions, using adapters from the MGIEasy PF Adapters-16 (Tube) Kit (MGI Tech). The concentration of each library was measured with a Qubit 3.0 Fluorometer using the dsDNA High-Sensitivity Assay Kit (Thermo Fisher Scientific, Waltham, MA, USA). The quality of each library was assessed using the Agilent 2100 Bioanalyzer with the High Sensitivity DNA Kit (Agilent Technologies). Circular DNA was generated using the MGIEasy Circularization Kit (MGI Tech) and DNA nanoballs were produced using the DNBSEQ-G400 RS High-Throughput Sequencing Kit (MGI Tech), in accordance with the manufacturer’s protocol. Sequencing was performed using the DNBSEQ-G400 platform (MGI Tech) in 2 × 150 bp. The resulting reads are available at the NCBI sequence read archive under project PRJNA1302757.

The 3′- and 5′-end adapter sequences were trimmed, and low-quality reads were removed using fastp (version 0.23.2) [20] with the following parameters “-q 20 -t 1 -T 1 -l 20 --adapter_sequence AAGTCGGAGGCCAAGCGGTCTTAGGAAGACAA and --adapter_sequence_r2 AAGTCGGATCGTAGCCATGTCGTTCTGTGAGCCAAGGAGTTG.”

### 2.3. Removal of Host-Derived Reads

After adapter trimming and quality filtering with fastp, host-derived reads were removed using KneadData (version 0.12.3) (https://huttenhower.sph.harvard.edu/kneaddata/, accessed on 25 December 2024), which screens reads against reference databases using Bowtie2 (version 2.5.4) [21]. We screened paired-end reads against (i) the *T. occipitalis* reference genome assembly reported by Kataoka et al. [22] (assembled genome sequence deposited in DDBJ BLKR01000001–BLKR01019865; BioProject PRJDB9056) and (ii) a human Bowtie2 database. Because trimming was already performed with fastp, the “--bypass-trim” option was used. KneadData was executed by providing both reference databases so that reads aligning to either reference were discarded. The complete bash script used in this study (including database download/indexing and command lines) is provided in Supplementary Materials (Script S1).

### 2.4. Assembly, Taxonomic Classification and Abundance Profiling

After data preprocessing, the remaining reads were de novo co-assembled into contigs using metaSPAdes (version 4.2.0) [23] with the default settings, and contigs shorter than 500 bp were excluded from downstream analyses. Retained contigs were clustered at 95% identity using CD-HIT-EST (version 4.8.1) [24] to remove redundant sequences. Taxonomic classification of these non-redundant contigs was performed using Kaiju (version 1.10.1) [25] against the NCBI non-redundant (nr) database (version 2024-08-25). Quality-filtered metagenomic reads from each sample were aligned to the contigs using Bowtie2 (version 2.5.4) [21], and the resulting SAM files were converted into BAM files using SAMtools (version 1.19.2) [26]. Relative taxonomic abundances were calculated as transcripts per million (TPM) from the BAM files using CoverM (version 0.7.0) [27].

### 2.5. Gene Prediction and Functional Profiling

Open reading frames (ORFs) were predicted from the assembled contigs using Prodigal (version 2.6.3) [28] in metagenomic mode, and ORFs shorter than 100 nucleotides were filtered. To construct a non-redundant gene catalog, all predicted protein sequences were clustered with CD-HIT (version 4.8.1) [24] using a 95% identity and 90% coverage, and the longest sequence from each cluster was selected as the representative sequence. Read mapped to the predicted ORFs were counted using featureCounts (version 2.1.1) [29] with BAM and GFF files generated by Prodigal. The resulting raw read counts were then normalized to TPM. Non-redundant gene catalogs were annotated against the NCBI COG 2024 database [30] using DIAMOND BLASTp (version 2.1.12) [31] and against the KEGG database (release 111.0) with KofamScan (version 1.3.0) using the prokaryotic HMM profiles (profile prokaryote.hal) [32], both of which were performed using default parameters. For each KEGG Orthology (KO) and Clusters of Orthologous Groups (COG), abundance was calculated by summing the abundances of all assigned genes.

### 2.6. Hierarchical Clustering and Heatmap Visualization

Hierarchical clustering of bacterial community profiles was conducted in R (version 4.3.3) [33] using the ComplexHeatmap package (version 2.18.0) [34]. To minimize the influence of rare taxa, only phyla, families, genera, and species with a relative abundance ≥1% in at least one sample were included in the hierarchical clustering analysis of the bacterial composition. The resulting relative-abundance matrix was row-wise Z-score-standardized, after which Euclidean distances were calculated with the “dist” function from the stats package (version 4.3.3). Hierarchical clustering was performed with the complete linkage method using the “hclust” function, and dendrograms were visualized with ComplexHeatmap.

### 2.7. Rarefaction, Alpha-Diversity, and Beta-Diversity Analyses

To evaluate community diversity, rarefaction curves based on detected species richness, KOs and COGs were generated. Alpha-diversity metrics (Chao1 [35], Shannon [36], Simpson [37], and Pielou’s evenness [38]) were calculated, and group differences were tested using pairwise Wilcoxon rank-sum tests. Beta-diversity was quantified using Bray–Curtis distances [39] and visualized using principal coordinate analysis (PCoA). Rarefaction, alpha-diversity, and beta-diversity analyses were performed in R using the vegan package (version 2.6.10) [40].

### 2.8. Differential Analysis of Bacterial Composition and Functional Potential

Differential abundance analysis of bacterial composition was conducted with DESeq2 (version 1.42.1) [41] at the phylum, family, genus, and species levels, as recommended in benchmark papers [42,43]. Library-size normalization was performed using the poscounts method, and pairwise contrasts were specified to identify taxa that differed significantly among females from mixed-sex rearing, females from female-only rearing, and males. To reduce noise due to sparse counts, we retained only taxa with a mean relative abundance >0.05% in at least one experimental group. Subsequently, DESeq2 was applied to the filtered raw-count matrix.

Functional gene abundance was analyzed with the same statistical framework. Genes with an average abundance > 1 TPM in any group were retained, after which DESeq2 was applied to the corresponding raw counts. *p*-values were adjusted using the Benjamini–Hochberg procedure implemented in DESeq2, and results with an adjusted *p*-values < 0.05 were considered statistically significant.

## 3. Results

### 3.1. Composition of the Gut Microbiome in the Field Cricket T. occipitalis

We analyzed the gut microbiomes of 45 adult crickets across three experimental groups: females from mixed-sex rearing, females from female-only rearing, and males (*n* = 15 per group). Our main comparison was between the two female groups. Because our prior work suggested sex-specific gut microbiomes [16], we also compared females and males within the mixed-sex rearing condition to examine sex-related variation. For each group, we generated three biological replicates by pooling five individuals per replicate. Shotgun metagenomic sequencing generated about 703.6 million raw reads, of which about 290.7 million were microbial reads (41.3% of the total) after quality control and host genome removal (Table S1). De novo co-assembly of all samples produced a reference gut microbiome comprising 232,128 contigs totaling 1.23 Gbp with an N50 of 42.4 Kbp (Table S2).

Taxon abundances were calculated by integrating taxonomic assignments with TPM values for all contigs. Rarefaction curves for species richness, KO groups, and COGs approached saturation, confirming an adequate sequencing depth for comprehensive diversity profiling (Figure S1).

Gut microbial contigs were classified into three major domains: bacteria, viruses, and archaea. In all samples, the proportions of these categories were approximately 97.6%, 1.4%, and 0.2%, respectively, closely mirroring the pattern previously reported for *Gryllotalpa orientalis* Burmeister (Orthoptera: Gryllotalpidae), a related species within the same infraorder (Table S3) [44].

**Figure 1:**
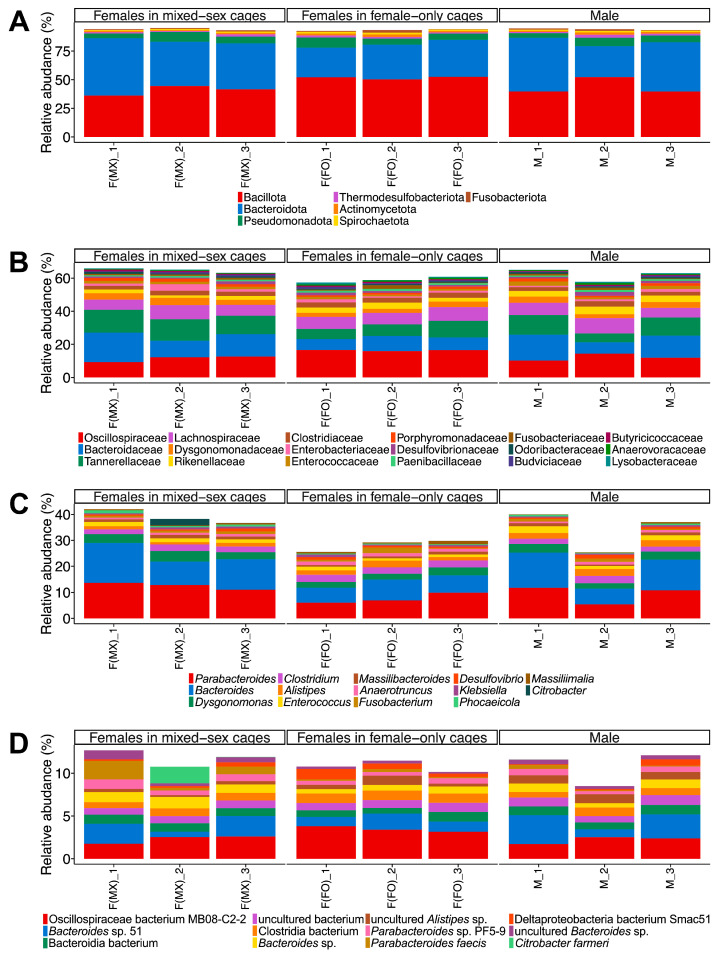
Taxonomic composition of gut bacterial communities in *T. occipitalis*. Relative abundances of bacterial communities are presented at (**A**) phylum, (**B**) family, (**C**) genus, and (**D**) species levels across three experimental groups: females from mixed-sex rearing (F(MX)), females from female-only rearing (F(FO)), and males (M). Each bar represents one biological replicate (*n* = 3 per group). Taxa that represent ≥1% of the community in at least one sample are shown for all samples, whereas taxa below 1% in all samples are not displayed.

The bacterial community encompassed 125 phyla, 643 families, 2606 genera, and 9331 species (Figure 1 and Table S4). At the phylum level, the gut microbiome was dominated by Bacillota, Bacteroidetes, and Pseudomonadota (Figure 1A). This finding is consistent with previous 16S rRNA gene amplicon sequencing studies on *T. occipitalis* (Serville), *T. oceanicus* (Le Guillou), and the spring field cricket *Gryllus veletis* (Alexander & Bigelow) (all Orthoptera: Gryllidae) [16,18,45]. At the family level, Oscillospiraceae, Bacteroidaceae, and Tannerellaceae were among the most abundant taxa across all groups (Figure 1B). At the genus level, *Parabacteroides*, *Bacteroides*, and *Dysgonomonas* were consistently dominant, and the species-level composition revealed that several taxa, including Oscillospiraceae bacterium MB08-C2-2 and *Bacteroides* sp. 51, were the most prevalent (Figure 1C,D). The virome was dominated by Uroviricota and Caudoviricetes, while the archaeome was dominated by Euryarchaeota (Figure S2, Tables S5 and S6). These patterns mirror those reported in other insects, such as the honey bee *Apis mellifera* Linnaeus [46], the Formosan subterranean termite *Coptotermes formosanus* Shiraki [47], and related crickets [44].

### 3.2. Gut Microbiome Differences Across Social Rearing Regimes in Female T. occipitalis

**Figure 2:**
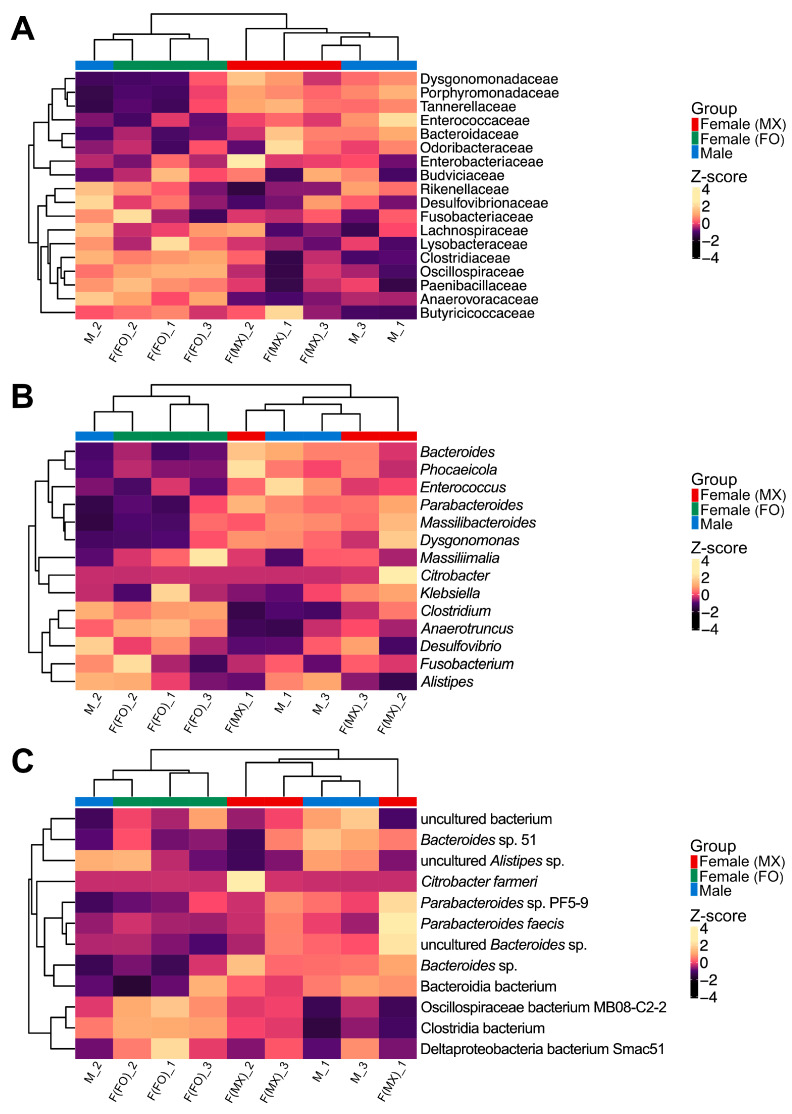
Hierarchical clustering analysis of gut bacterial communities. Heatmaps showing clustering patterns of bacterial relative abundances at (**A**) family, (**B**) genus, and (**C**) species levels across three experimental groups: females from mixed-sex rearing (F(MX)), females from female-only rearing (F(FO)), and males (M). Each column represents one biological replicate. Colors represent Z-score normalized abundances calculated per taxon across all samples, with lighter colors indicating higher relative abundances. Column annotation bars identify the experimental groups, with red indicating females from mixed-sex rearing, green indicating females from female-only rearing, and blue indicating males. Taxa that represent ≥1% of the community in at least one sample are shown for all samples, whereas taxa below 1% in all samples are not displayed.

**Figure 3:**
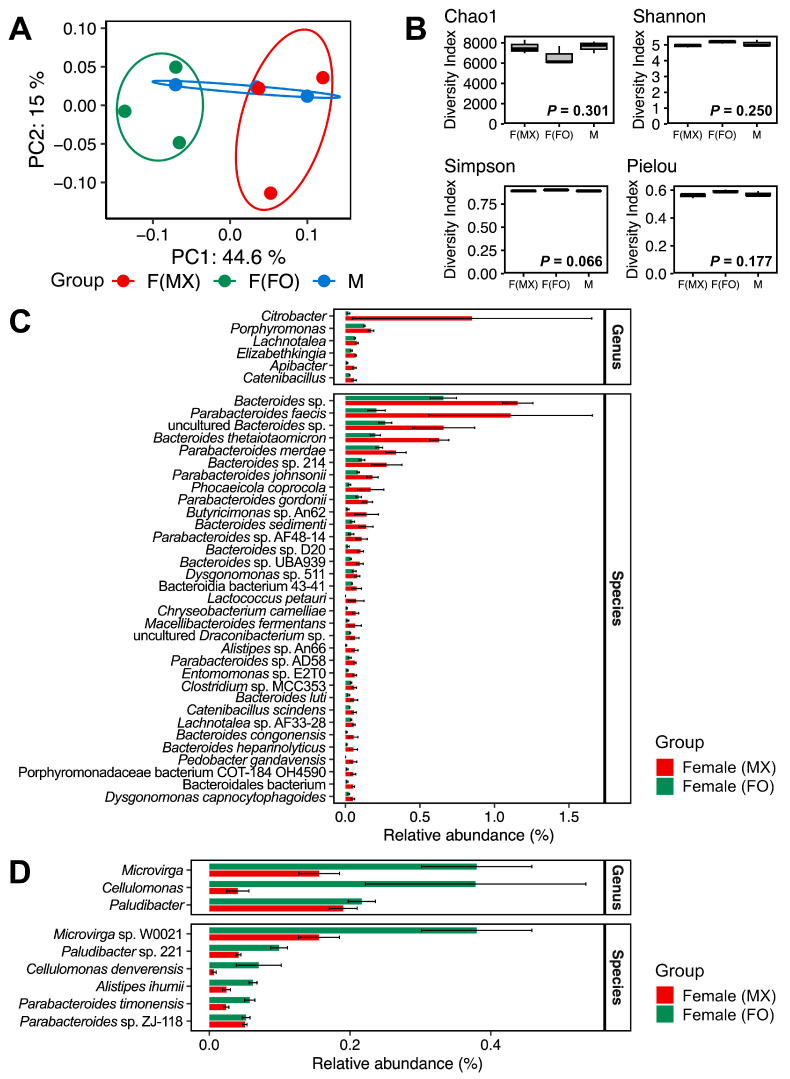
Gut microbiome diversity and composition across experimental groups. (**A**) PCoA of Bray–Curtis dissimilarities with 95% confidence ellipses. Red represents females from mixed-sex rearing, green represents females from female-only rearing, and blue represents males. (**B**) Alpha-diversity indices (Chao1 richness, Shannon diversity, Simpson diversity, and Pielou evenness) for females from mixed-sex rearing (F(MX)), females from female-only rearing (F(FO)), and males (M). (**C**) Relative abundances of gut bacterial genera and species enriched in females from mixed-sex rearing compared with females from female-only rearing. (**D**) Relative abundances of gut bacterial genera and species enriched in females from female-only rearing compared with females from mixed-sex rearing. Bars show means ± standard errors. Displayed taxa are genera and species that differed significantly in DESeq2 analysis (FDR-adjusted *p* < 0.05, Benjamini–Hochberg correction). These samples were taken from the females from mixed-sex rearing (F(MX)) and females from female-only rearing (F(FO)), displayed in red and green, respectively.

To examine how gut microbiome composition covaries with social rearing regimes in females, we employed multiple complementary approaches. Hierarchical clustering with Euclidean distances revealed distinct grouping patterns in gut microbiome composition. Across all taxonomic levels (family, genus, and species), females from mixed-sex and female-only rearing formed different clusters (Figure 2). In contrast, the male replicates did not form a single cohesive cluster. One male sample was grouped with the female-only cluster, whereas the other two were grouped with the mixed-sex female cluster (Figure 2). This pattern suggests that male gut community profiles overlapped with both female groups and showed higher among-replicate variability. This pattern was also evident in the PCoA, which suggested separation between females from mixed-sex and female-only rearing (Figure 3A). No significant differences were observed in alpha-diversity indices among the groups (Kruskal–Wallis test: Chao1, *p* = 0.301; Shannon, *p* = 0.250; Simpson, *p* = 0.066; Pielou, *p* = 0.177) (Figure 3B and Table S7).

Using differential abundance analysis with DESeq2, we identified the specific taxa associated with these compositional differences. Females from mixed-sex rearing showed enrichment of six genera and 33 species, including *Lactococcus* (Figure 3C), consistent with findings from the Mormon cricket *A. simplex* (Haldeman), where females without mating opportunities were found to harbor fewer lactic-acid bacteria [8]. In contrast, females from female-only rearing were enriched in three genera (*Microvirga*, *Cellulomonas*, and *Paludibacter*) and six species (*Microvirga* sp. W0021, *Paludibacter* sp. 221, *Cellulomonas denverensis*, *Alistipes ihumii*, *Parabacteroides timonensis*, and *Parabacteroides* sp. ZJ-118) (Figure 3D).

In light of evidence from our prior work suggesting sex effects in the gut microbiome [16], we also examined sex differences under mixed-sex rearing. Comparisons between males and females from mixed-sex rearing revealed genus-level differences (five genera enriched in males and two in females), but no species-level differences (Figure S3A,B and Table S8).

Taken together, these results indicate that female gut microbiome composition was consistently associated with the rearing regime in our dataset, with females from mixed-sex and female-only rearing showing consistent differences. These patterns suggest that the social rearing regime may represent an important axis of variation in the hindgut microbiome of *T. occipitalis*.

### 3.3. Functional Analysis of Gut Microbiome Metabolic Potential Using KEGG Orthology (KO)

To assess functional differences in metabolic potential, we annotated the predicted genes against the KO database and identified 5944 KOs across all samples. Under the same comparative conditions described in the previous section, we performed a differential abundance analysis using DESeq2 for these KOs to test for the differences between the experimental groups.

**Figure 4:**
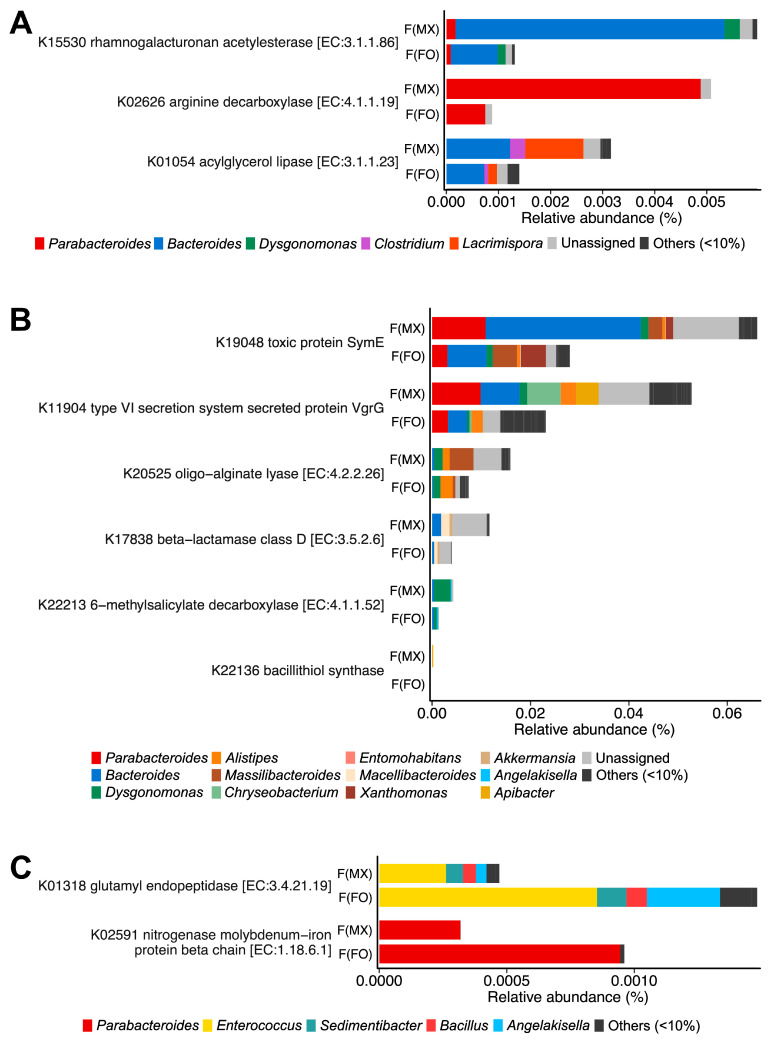
Functional gene differences between experimental groups using KEGG Orthology. Differential abundance of KEGG Orthologs between females from mixed-sex rearing (F(MX)) and females from female-only rearing (F(FO)). (**A**) Genes involved in nutrient catabolism. (**B**) Genes linked to interference competition. (**C**) Genes encoding glutamyl endopeptidase and nitrogenase. Bars are colored by genus-level contributions. Taxa contributing ≥10% in at least one gene are shown, and KEGG orthologs with significant differences in DESeq2 analysis (FDR-adjusted *p* < 0.05, Benjamini–Hochberg correction) are displayed.

Females from mixed-sex rearing showed higher relative abundances of 27 KOs, many of which were annotated to pathways related to nutrient catabolism and interbacterial interactions. Key functions included polysaccharide, arginine, and acylglycerol degradation (K15530, K02626, and K01054) (Figure 4A and Table S9), as well as functions linked to bacterial cooperation and antagonism, including type VI secretion and toxin–antitoxin systems (K19048, K11904, K20525, K17838, and K22213) (Figure 4B and Table S9). These functions were predominantly contributed by *Bacteroides*, *Parabacteroides*, and *Dysgonomonas* (Figure 4A,B). In contrast, females from female-only rearing were enriched in 14 KOs, including glutamyl endopeptidase (K01318) and a KO annotated as nitrogenase (K02591) (Figure 4C and Table S9), mainly assigned to *Parabacteroides* and *Enterococcus*.

These KO-based patterns suggest that the gut microbiomes of females from mixed-sex rearing had a higher relative enrichment of genes annotated to nutrient degradation and interbacterial competition, whereas those from female-only rearing had a higher relative enrichment of genes annotated as involved in metabolic self-reliance and extracellular proteolysis. Additionally, no functional differences were observed between males and females under mixed-sex rearing (Table S9).

### 3.4. Functional Analysis Using Clusters of Orthologous Groups (COGs)

To complement the KEGG analysis, we also annotated the predicted genes against the COG database, identifying 5050 COGs across all samples. Differential abundance analysis using DESeq2 revealed functional patterns consistent with the KEGG results.

**Figure 5:**
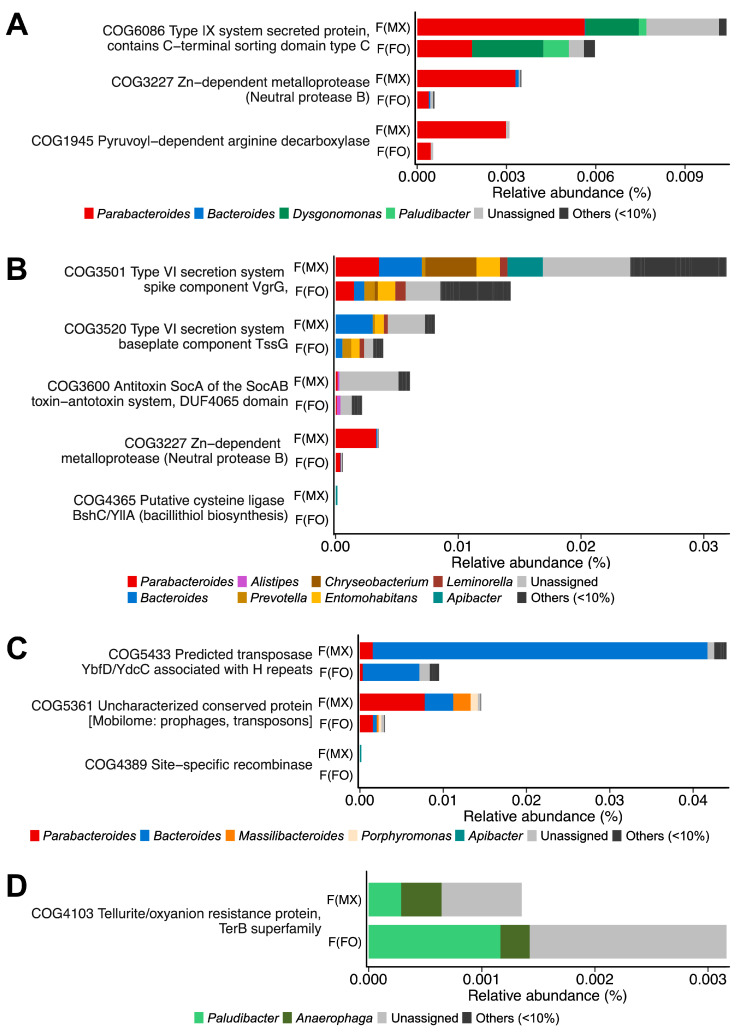
COG-based functional profiling of gut microbiome differences. Differential abundance of COG categories between females from mixed-sex rearing (F(MX)) and females from female-only rearing (F(FO)). (**A**) Nutrient-catabolism genes, (**B**) genes related to interference competition, (**C**) genes linked to mobile element genes, and (**D**) genes encoding a tellurite/oxyanion resistance protein. Bars are colored by genus-level contributions. Taxa contributing ≥10% in at least one gene are shown, and COGs with significant differences in DESeq2 analysis (FDR-adjusted *p* < 0.05, Benjamini–Hochberg correction) are displayed.

Females from mixed-sex rearing showed 21 enriched COGs associated with dietary fiber and arginine/protein catabolism (COG6086, COG3227, and COG1945) (Figure 5A and Table S9), bacterial competition mechanisms (COG3501, COG3520, COG3600, COG3227, and COG4365) (Figure 5B and Table S9), and mobile genetic elements facilitating horizontal gene transfer (COG5433, COG5361, COG4389) (Figure 5C and Table S9). These enriched COG categories were mainly contributed by *Bacteroides*, *Parabacteroides*, and *Dysgonomonas* (Figure 5A–C). In contrast, females from female-only rearing were enriched in 11 COGs related to tellurite/oxyanion resistance (COG4103), mainly contributed by *Paludibacter* and *Anaerophaga* (Figure 5D and Table S9).

Overall, these COG-based results were broadly consistent with the KO-based patterns, suggesting that the social rearing regime is associated with differences in the prevalence of genes linked to nutrient breakdown, interbacterial competition, mobile genetic elements, and oxidative stress defense in the female gut microbiome. Additionally, under mixed-sex rearing, males showed a higher relative abundance of genes annotated as the uncharacterized protein *YvbJ* (COG4640) than females, with annotations primarily assigned to taxa including *Enterococcus*, *Ruminococcus*, and *Alkalibacterium* (Figure S4 and Table S9).

## 4. Discussion

Crickets are valuable models for studying insect physiology and behavior, and are also gaining importance as a sustainable source of protein [11]. Understanding how social environments relate to gut microbiome variation is important for generating hypotheses about the mechanisms underlying behavioral and physiological plasticity in insects. Here, using shotgun metagenomic sequencing, we characterized the taxonomic composition and functional gene repertoire of the hindgut microbiome of female field crickets *T. occipitalis* (Serville) under two social conditions: mixed-sex rearing and female-only rearing. This study provides a genome-resolved description of gut microbiome differences across social rearing regimes in female crickets, identifying specific taxonomic and functional shifts that may inform hypotheses about links to host behavioral and physiological plasticity. The findings in the present study are based solely on functional interpretations derived from metagenomic gene-content annotations. Therefore, we interpret the following statements cautiously because we did not quantify gene expression or microbial products.

Our analyses showed that females from mixed-sex rearing harbored gut bacterial communities distinct from those of females from female-only rearing, as supported by clustering, β-diversity, and differential abundance analyses. Similar associations between female social context (e.g., mating status or male contact) and gut microbiome composition have been reported in other insects, including the fall armyworm *S. frugiperda* (Smith) and the Mormon cricket *A. simplex* (Haldeman) [7,8]. Together, these findings suggest that the female gut microbiome can vary across social contexts that differ in male exposure and/or mating opportunities across multiple insect taxa, although the underlying drivers may differ among species.

Within mixed-sex rearing, differences in gut microbiome between the sexes were limited to modest genus-level shifts, with no consistent species-level or functional divergence. The only notable difference was a male-biased increase in the relative abundance of genes annotated as the uncharacterized protein *YvbJ* (COG4640). In contrast, females from mixed-sex and female-only rearing showed clearer separation in community composition. In our dataset, hindgut microbiome profiles clustered more clearly by social rearing regime than by sex. This pattern does not contradict prior work reporting limited sex-associated differences in the hindgut of this species [16].

Functional profiling further showed that female gut microbiomes differed in the relative representation of gene categories across social rearing regimes. Females from mixed-sex rearing showed higher relative abundances of genes annotated to polysaccharide, arginine, and lipid catabolism, along with an increased abundance of proteases and arginine decarboxylase. This pattern is consistent with the idea that differences in host traits related to the social environment, such as feeding behavior, may be reflected in the functional potential of the gut microbiome. For example, increased food intake following male contact has been reported in the short-tailed cricket *Anurogryllus muticus* (De Geer) [13]. Conversely, females from female-only rearing showed relative enrichment of genes annotated to nutritional stress tolerance, including nitrogenase [48], which could be consistent with reduced feeding reported for females without mating opportunities in *Gryllus assimilis* (Fabricius) [14]. Together, these annotations support the hypothesis that microbiome functional profiles may covary with female physiological state under different social rearing regimes.

In parallel with these potential metabolic differences, females from mixed-sex rearing also showed a relative enrichment of genes annotated to interbacterial competition in the female gut microbiome, particularly those encoding type VI secretion and toxin–antitoxin systems. This shift may reflect two key processes: horizontal microbial transmission during mixed-sex social interactions [9], and immune suppression associated with mating and/or male exposure in females, as documented in the bush cricket *Kawanaphila nartee* Rentz [49], the house cricket *Acheta domesticus* (Linnaeus) [12], and the dengue mosquito *A. aegypti* (Linnaeus) [6]. Reduced host immunity allows for increased bacterial density [6], which could intensify interference competition and favor bacteria equipped with competitive mechanisms [50]. Concurrent enrichment of mobile genetic elements suggests enhanced genomic plasticity, likely enabling the rapid acquisition of defense genes that provide competitive advantages [51,52]. Additionally, type VI secretion system-rich bacterial communities may protect the host by eliminating potential pathogens, as observed in mammalian systems [53]. Thus, the enrichment of type VI secretion system-related genes in females from mixed-sex rearing raises the possibility that competitive interactions could influence community stability and potentially pathogen susceptibility.

Females from female-only rearing showed a contrasting pattern, with gut microbiomes enriched for stress-resistance genes, including the *ter* gene cluster (*terB* superfamily) and glutamyl endopeptidase. One possible explanation is that these enrichments are consistent with differences in baseline antimicrobial activity that have been reported across female social contexts in other insects. In several insects, mating and/or social context can be associated with downregulation of immune-related pathways and antimicrobial defenses [49,54], whereas females under female-only rearing may maintain higher baseline antimicrobial activity, including reactive oxygen species (ROS) production and antimicrobial peptides. Such differences could create gut conditions that select for bacteria with oxidative stress tolerance (ter gene cluster) [55] and degrading host antimicrobial peptides (glutamyl endopeptidase) [56]. Supporting this interpretation, in *Drosophila* females, mating has been shown to reduce expression of NADPH oxidases, enzymes that generate reactive oxygen species (ROS) [57]. Similarly, in the dengue mosquito *A. aegypti* (Linnaeus), mating has been shown to suppress antimicrobial-peptide gene transcription in the gut [6].

This study has several limitations that should be considered when interpreting the results. First, our rearing regimes differed in multiple social components: mixed-sex cages allowed male exposure and potential mating, whereas female-only cages did not. Because mating status and male cues were not independently controlled, we cannot disentangle potentially confounding factors, such as mating effects versus non-mating effects of male exposure. Disentangling these components will require designs that control mating and male-derived cues independently (e.g., controlled mating and/or barrier cages). Putative functional shifts were inferred from gene-abundance profiles rather than direct measurements of microbial activity (e.g., gene expression or metabolites), so we cannot determine whether the observed gene-content patterns translate into functional changes or causal effects on host phenotypes. Our pooled replicates were limited, which may reduce statistical power, limit the precision of effect-size estimates, and affect the generalizability of the findings.

Within these constraints, we observed consistent differences in the taxonomic composition and functional gene profiles of the female gut microbiome between mixed-sex and female-only rearing under group-rearing conditions in our dataset. Our inferences were based solely on gene abundance patterns rather than on direct measurements of gene expression or microbial metabolites. Future studies integrating metatranscriptomics and metabolomics will be essential to determine whether the gene-content differences reported here are accompanied by corresponding changes in microbial transcription and metabolite outputs, thereby validating the functional significance of the inferred pathways and clarifying links to host phenotypes. Our dataset provides a genome-resolved baseline for how female gut microbiomes can vary across social rearing regimes and generates testable hypotheses for future work.

## Data Availability

All data generated or analyzed during this study are included in this published article and its supplementary information files. Raw sequence data were deposited in the NCBI BioProject (PRJNA1302757), BioSample (SAMN50491110–SAMN50491118), and SRA (SRR34918247–SRR34918249, SRR34922024–SRR34922029).

## Supplementary Materials

The following supporting information can be downloaded at: https://www.mdpi.com/article/10.3390/insects17010091/s1, Script S1: Bash workflow for removal of host-derived reads from shotgun metagenomes using KneadData (Bowtie2-based); Figure S1: Rarefaction curves based on the number of species, KOs, and COGs per sample for females from mixed-sex rearing (F(MX)), females from female-only rearing (F(FO)), and males (M); Figure S2: Gut virome and archaeome composition of *T. occipitalis*: females from mixed-sex rearing (F(MX)), females from female-only rearing (F(FO)), and males (M). Relative abundances are shown for four levels: (A) virome at the phylum level, (B) virome at the species level, (C) archaeome at the phylum level, and (D) archaeome at the species level. Taxa that represent ≥3% of the community in at least one sample are shown for all samples, whereas taxa below 3% in all samples are not displayed; Figure S3: Differences in the relative abundances of gut bacteria between females from mixed-sex rearing (F(MX)) and males (M). (A) Relative abundances of gut bacterial genera enriched in males compared with females from mixed-sex rearing. (B) Relative abundances of gut bacterial genera enriched in females from mixed-sex rearing compared with males. Bars show means ± standard errors. Taxa displayed are genera that differed significantly according to DESeq2, with *p*-values adjusted by the Benjamini–Hochberg procedure (FDR-adjusted *p* < 0.05). Red represents females from mixed-sex rearing and blue represents males; Figure S4: Differences in COG abundance and their genus-level contributors between females from mixed-sex rearing (F(MX)) and males (M). Taxa contributing ≥10% in at least one gene are shown, and COGs with significant differences in DESeq2 analysis (FDR-adjusted *p* < 0.05, Benjamini–Hochberg correction) are displayed; Table S1: Summary of metagenomic sequencing statistics for all samples across three experimental groups: females from mixed-sex rearing (F(MX)), females from female-only rearing (F(FO)), and males (M); Table S2: General statistics for de novo co-assembly of all samples; Table S3: Relative abundance table in domain levels for all samples; Table S4: Relative abundance table of bacterial composition; Table S5: Relative abundance table of viral composition; Table S6: Relative abundance table of archaeal composition; Table S7: Raw data and test results for alpha- and beta-diversity; Table S8: Results of DESeq2 analysis of differential relative abundances of gut bacteria; Table S9: Results of DESeq2 analysis of differential relative abundances of KOs and COGs.
