# Supplementary figures and images for: Gut Microbiome Differences Across Mixed-Sex and Female-Only Social Rearing Regimes in Female Field Crickets Teleogryllus occipitalis (Orthoptera: Gryllidae)
Source: Insects. 2026 Jan 13;17(1):91. doi: 10.3390/insects17010091 (PMC12841673; doi:10.3390/insects17010091)

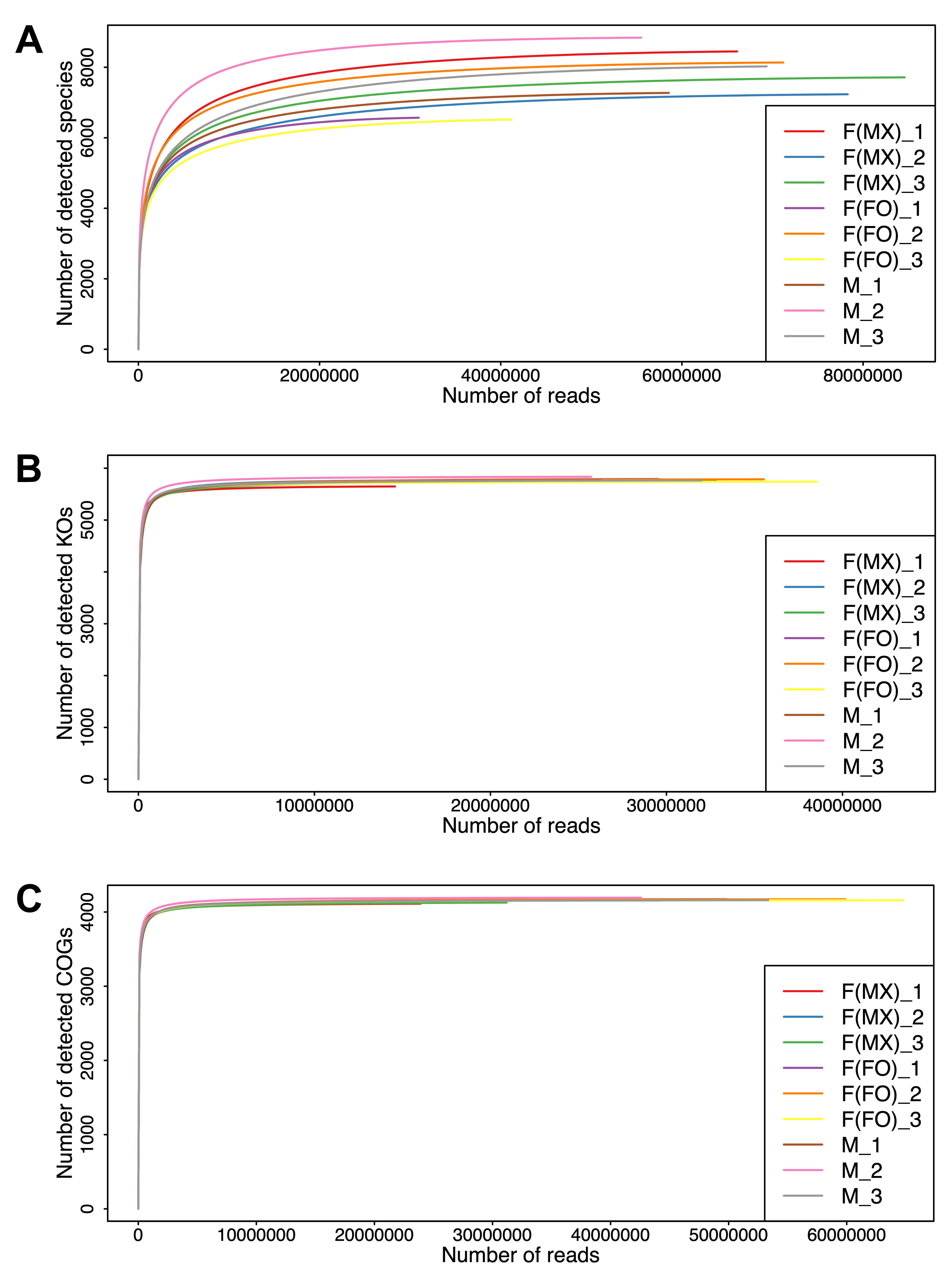

Supplement: Supplementary file 1 [file insects-17-00091-s001.zip › Figure S1_ver2.jpg]

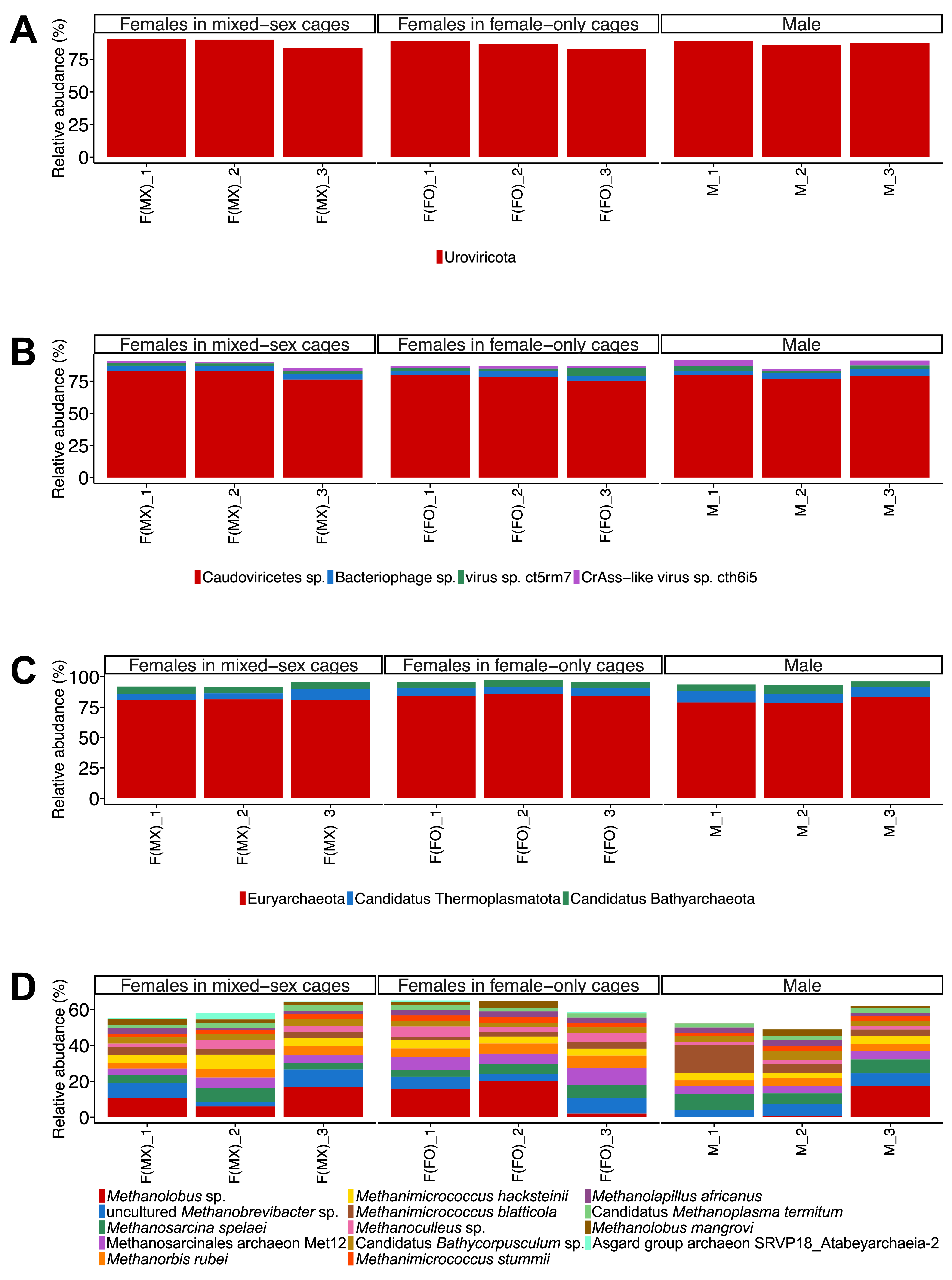

Supplement: Supplementary file 1 [file insects-17-00091-s001.zip › Figure S2_ver2.jpg]

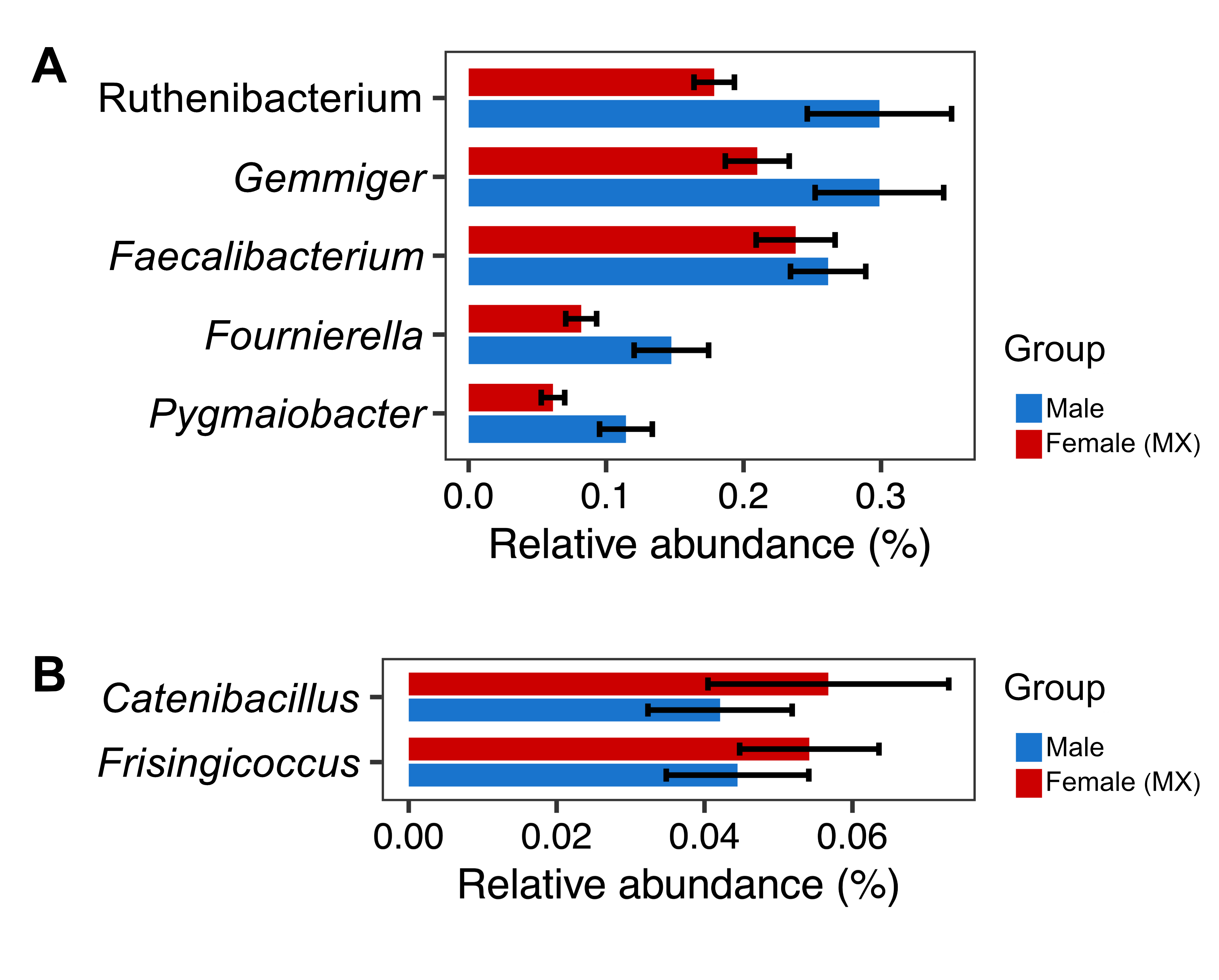

Supplement: Supplementary file 1 [file insects-17-00091-s001.zip › Figure S3_ver2.jpg]

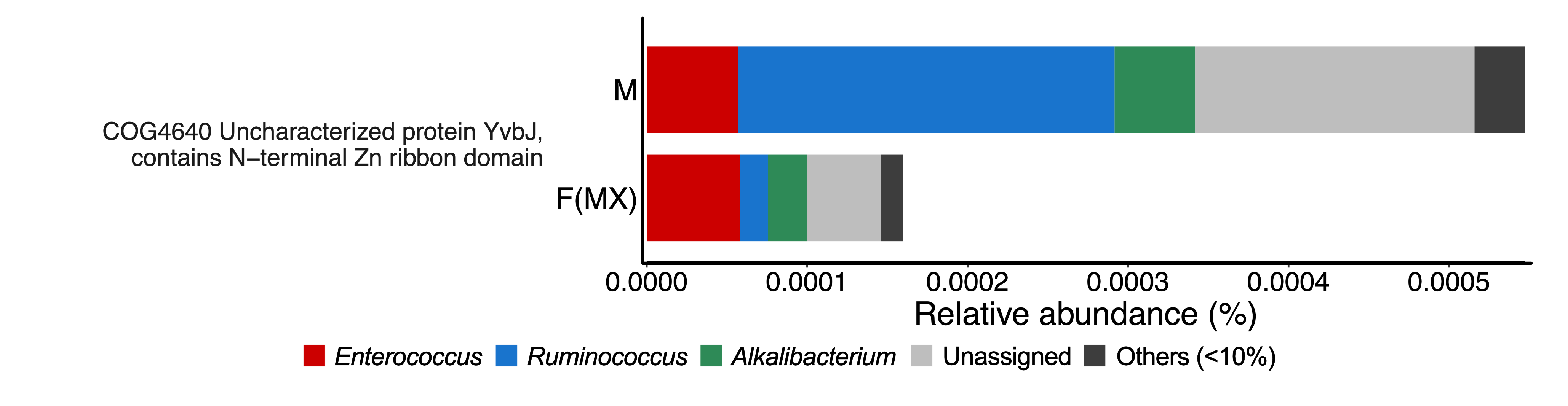

Supplement: Supplementary file 1 [file insects-17-00091-s001.zip › Figure S4.jpg]
